# Aplastic anemia and paroxysmal nocturnal hemoglobinuria in children and adults in two centers of Northern Greece

**DOI:** 10.3389/fonc.2022.947410

**Published:** 2022-11-10

**Authors:** Eleni Gavriilaki, Athanasios Tragiannidis, Maria Papathanasiou, Sotiria Besikli, Paraskevi Karvouni, Vassiliki Douka, Eleni Paphianou, Emmanuel Hatzipantelis, Giorgos Papaioannou, Anastasia Athanasiadou, Anastasia Marvaki, Alkistis-Kira Panteliadou, Anna Vardi, Ioannis Batsis, Antonia Syrigou, Despina Mallouri, Chrysavgi Lalayanni, Ioanna Sakellari

**Affiliations:** ^1^ Hematology Department and Bone Marrow Transplant (BMT) Unit, G Papanicolaou Hospital, Thessaloniki, Greece; ^2^ 2^nd^Paediatric Department, American Hellenic Educational Progressive Association (AHEPA) Hospital, Aristotle University of Thessaloniki, Thessaloniki, Greece; ^3^ School of Medicine, University of Thessaloniki, Thessaloniki, Greece

**Keywords:** bone marrow failure (BMF), bone marrow failure syndromes (BMFs), paroxysmal nocturnal hemoglobinuria, hematopoietic (stem) cell transplantation (HCST), fanconi anaemia

## Abstract

Bone marrow failure (BMF) syndromes are a group of various hematological diseases with cytopenia as a main common characteristic. Given their rarity and continuous progress in the field, we aim to provide data considering the efficiency and safety of the therapeutic methods, focusing on the treatment of aplastic anemia(AA) and paroxysmal nocturnal hemoglobinuria (PNH). We enrolled consecutive patients diagnosed with BMF in two referral centers of Northern Greece from 2008 to 2020. We studied 43 patients with AA (37 adults and 6 children/adolescents) and 6 with classical PNH. Regarding classical PNH, 4 patients have received eculizumab treatment with 1/4 presenting extravascular hemolysis. Among 43 patients with aplastic anemia, PNH clones were detected in 11. Regarding patients that did not receive alloHCT (n=15), 14/15 were treated with ATG and cyclosporine as first line, with the addition of eltrombopag in patients treated after its approval (n=9). With a median follow-up of 16.7 (1.8-56.2) months from diagnosis, 12/14 (85.7%) are alive (4-year OS: 85.1%). AlloHCT was performed in 28 patients. Five patients developed TA-TMA which did not resolve in 3/5 (all with a pre-transplant PNH clone). With the follow-up among survivors reaching 86.3 (6.3-262.4) months, 10-year OS was 56.9%, independently associated with PNH clones after adjusting for age (p=0.024). In conclusion, our real-world experience confirms that novel treatments are changing the field of BMF syndromes. Nevertheless, there is still an unmet need to personalize algorithms in this field.

## Introduction

Bone marrow failure (BMF) syndromes are a group of various hematological diseases that have a main common characteristic: the cytopenia of one or more blood cell lines resulting in anemia, neutropenia and/or thrombopenia (1). Bone marrow failure syndromes can be divided in two main categories, acquired and congenital disorders (2). Acquired syndromes represent an abnormal immune response to an external factor such as infections mostly viral, drugs or chemicals and usually affect all three lines causing pancytopenia. On the other hand, inherited bone marrow failure syndromes (IBMF) occur due to mutations in the hematopoietic stem cell or other progenitor cells (3). The most common congenital disorders (1), eithercause pancytopenia such as Fanconi anemia (4) and dyskeratosis congenita (5) or primary affect one lineage such as Shwachman-Diamond syndrome (6), Diamond-Blackfan anemia (7), Kostmann syndrome and congenital amegakaryocytic thrombocytopenia (8). They also feature a predisposition for congenital malformations and progression to myelodysplasia, acute leukemias and solid tumors (9). Due to presentation variability increased awareness and continuous follow-up are always needed, even though acquired BMF syndromes are more frequent in both adults and children (10).

Aplastic anemia (AA) is the most common acquired BMF syndrome with an incidence of 2 per million in Western countries and up to 6 per million in Asia (11). AA is a diagnosis of exclusion with hypoplastic MDS and IBMF syndromes being the main conditions that need to be excluded especially in children aged <10 years. In favor of AA are the presence of peripheral pancytopenia, an ‘‘empty’’ bone marrow with a lack of dysplasia (12–14). In the context of acquired BMF syndromes, paroxysmal nocturnal hemoglobinuria (PNH) is a clonal disease caused by a somatic mutation in the PIGA-gene resulting in the deficiency of GPI-anchored proteins (such as CD55 and CD59) and leads to erythrocytes unable to control complement activation (15–18). This results in chronic intravascular hemolysis, thrombosis in unusual locations and BMF caused by cellular autoimmunity to HSCs (19–22). Apart from its classical form that is characterized by a cellular or even hypercellular marrow, a PNH clone can also be detected in up to 70% of patients with AA (23–25). In children, the percentage of PNH clone is much lower and varies between 21-53% according to different studies (26, 27). When the PNH clone increases, especially after immunosuppressive therapy, they may present with classic complications of PNH (28, 29).

There are two main upfront treatments for AA: the immunosuppressive treatment (IST), a choice suitable for patients older than 40 years, and allogeneic hematopoietic cell transplantation (HCT) for patients younger than 40 (30–32). The best cell source for HCT remains the bone marrow because it reduces the possibility of a Graft-versus-Host Disease (GVHD);the most suitable donor is a matched sibling donor (33–35). If a matched sibling donor isn’t available, a matched unrelated donor (UD) is searched, while other options are unrelated cord blood and haploidentical transplants (34, 36). HCT from UD can also be the frontline treatment for pediatric patients (younger than 20 years) (37). On the other hand, complement inhibitors are the main treatment for PNH. Eculizumab (a humanized monoclonal antibody against C5) has been the first-in-class inhibitor (38). Despite its benefits, new C5 inhibitors are being developed with the second-generation C5 inhibitors being approved, ravulizumaband crovalimab (a long acting anti-C5 monoclonal antibody) showing non-inferiority to eculizumab (39–42). The results from the development of upstream inhibitors with the C3 inhibitor pegcetacoplan receiving approval, and factors B and D being investigated within phase 3 registration trials are also encouraging (43–46).

Given the rarity of these entities and continuous progress in the field, we aim to provide data considering the efficiency and safety of the therapeutic methods, focusing on the treatment of AA and PNH.

## Methods

### Patient population

We enrolled consecutive patients diagnosed with BMF in two referral centers of Northern Greece from 2008 to 2020: AHEPA Hospital for the pediatric and adolescent population and Papanikolaou Hospital for the adolescent and adult population. Patients diagnosed with hypoplastic myelodysplastic syndrome (MDS) were excluded from the present study, in order to avoid heterogeneity in the study population. All patients were tested for PNH clones using a standardized flow cytometry protocol based on FLAER (fluorescent aerolysin) detection (47). Disease was treated according to ongoing recommendations during each treatment period (33). In particular, patients younger than 40 years with a sibling donor proceeded to upfront alloHCT. Patients older than 40 years, or without a sibling donor, immunosuppression was the first-line therapy. Refractory or relapsed patients proceeded to alloHCT if eligible and with a suitable donor. HLA typing was performed at diagnosis for all patients. Standard of care was similar to both centers, according to current guidelines.

This study was a retrospective chart review, and it was approved by the institutional review board and ethics committee of G. Papanicolaou Hospital. All patients gave written informed consent. The study was conducted in compliance with the Helsinki Declaration.

### Standard of care

BMF patients were admitted to neutropenic isolation rooms. According to ongoing protocols, patients received irradiated Red Blood Cell (RBC) and platelet transfusions only at a clinical indication (signs/symptoms of anemia or thrombocytopenia) or at Hemoglobin < 7 g/dl or platelet count < 10K/μL. GCSF (Granulocyte colony-stimulating factor) was administered in cases of persistent grade 4 neutropenia in patients with signs/symptoms of infection. Routine blood and urine cultures were performed once weekly in hospitalized patients. Wide spectrum antibiotics were administered according to ongoing protocols, with modification according to cultures. Prophylaxis for Pneumocystis jiroveci, herpes simplex, and Candida spp were also administered in hospitalized and neutropenic outpatient setting. Patients with PNH received prophylactic anticoagulation as standard practice. Prophylaxis was given to all patients, even those without thrombosis and was stopped after initiation of complement inhibition.

### HCT standards of practice

Conditioning regimens included Cyclophosphamide (50 mg/kg/day for 4 days) and Antithymocyte Globulin (rabbit ATG, Thymoglobulin 2.5 mg/kg/day for 3 days). In patients sensitized with multiple transfusions (48), modifications were performed accordingly: Cyclophosphamide (50 mg/kg/day for 4 days), Fludarabine (30 mg/m2/day for 4 days), ATG 7.5 mg/kg and 10 mg/kg for sibling and unrelated donors respectively, as previously described (49). GVHD prophylaxis consisted of Methotrexate and Cyclosporine. Cyclosporine was slowly tapered and stopped between 9-12 months post-transplant with a careful follow-up of blood counts. STR (short tandem repeat) fragment analysis was performed regularly (on day + 14, + 30, + 60, + 90) in unfractionated bone marrow for chimerism evaluation. Complete donor chimerism was defined as donor chimerism ≥99% (50). Regarding supportive care, patients were admitted to neutropenic isolation rooms with HEPA filters. Prophylaxis for Pneumocystisjiroveci, herpes simplex, and Candida spp was administered. Patients underwent Cytomegalovirus (CMV) and Epstein-Barr (EBV) surveillance using peripheral blood molecular assays (51).

### Statistical analysis

Analysis was performed with SPSS 22.0 (IBM SPSS Statistics for Windows, Version 22.0. Armonk, NY: IBM Corp). The following demographic and laboratory/clinical factors were included: transfusions (red cells, platelets), pre-transplant lines of treatment, age, gender, hemoglobin, neutrophils, and platelets values at diagnosis, PNH clone. Additionally, transplant factors were registered: donor type (sibling/unrelated), graft source (bone marrow/peripheral), HLA matching, conditioning (Cyclophosphamide/ATG with/without fludarabine); post-transplant factors: neutrophil and platelet engraftment, severe acute (grade 2-4) and extensive chronic GVHD, TA-TMA (transplant-associated thrombotic microangiopathy), PTLD (Post-transplant lymphoproliferative disease), infections, late complications, relapse, and survival. Chi-square test, Student’s t-test or Mann-Whitney test were used to compare variables. Overall survival (OS) probability was calculated with Kaplan-Meier curves. Variables with p<0.1 in univariate analysis were included in multivariate analysis using Cox proportional hazards. Cumulative incidence of competing events analysis was calculated by the EZR software (52). Statistical significance was assessed by the Gray test and Fine and Gray regression modeling. Significance level was 0.05 and two-tailed.

## Results

### Patient population

In total, we examined43 patients with severe or very severe AA (37 adults and 6 children/adolescents, **Table 1**) and 6 adult patients with classical PNH. Regarding cytogenetics, bone marrow samples failed to yield metaphases in 12 patients and normal cytogenetics were detected in 26patients. Cytogenetic abnormalities were detected in 11patients: trisomy 6 in 6 patients, trisomy 8 in 4, andmonosomy 7 in 1 patient.

**Table 1:** Baseline patients’ characteristics for AA patients (n=43).

Characteristics	HCT (n=28)	no-HCT (n=15)	p-value
**Age, years**	23.3 (20.1)	34.4 (42.0)	0.130
**Male gender, n (%)**	17 (58)	10 (67)	0.130
**Severe:very severe AA, n (%)**	21 (72): 8 (28)	12 (80): 3 (20)	0.582
**ARC (10^4^/μL)**	2.4 (1.3)	2.5 (1.2)	0.682
**Hemoglobin at diagnosis, g/dl**	9 ± 1	7 ± 2	0.029
**Neutrophils at diagnosis (x10^9^/L)**	0.8 (1.0)	0.4 (1.0)	0.536
**Platelets at diagnosis (K/μL)**	22.0 (19.2)	19.0 (3.8)	0.634
**PNH clone at diagnosis, n (%)**	4 (14)	7 (48)	0.017

AA, aplastic anemia; PNH, Paroxysmal nocturnal hemoglobinuria. ARC, absolute reticulocyte count. Continuous variables are presented as median (interquartile range).

Regarding patients with classical PNH, 4 patients have received eculizumab treatment for a median of 5.1 years (range 2.1-8.2). Among them, 3 out of 4 have shown hemoglobin normalization and no transfusion requirements, while the fourth patient presented with extravascular hemolysis and regular transfusion requirements. No adverse event related to eculizumab was noted. Three patients remain under eculizumab treatment. Furthermore, 2 patients received crovalimab under the COMMODORE-1/2 open-label clinical trials: one switched from eculizumab (NCT04434092) and the other one started as a naïve patient (NCT04432584). One patient with classical PNH and history of thrombosis has never consented to receive complement inhibitors and remains with supportive treatment.

#### Aplastic anemia

Among 43 patients with aplastic anemia, PNH clones were detected in 11 patients (median 4%, range 1-65%), of whom, 4 patients did not proceed to alloHCT. Only one patient was treated with eculizumab, two years after immunosuppressive therapy, due to prominent hemolytic anemia attributed to a PNH clone larger than 30% in neutrophils (65%). The patient achieved hemoglobin normalization. **Table 1** summarizes baseline characteristics in patients that received alloHCT compared to those who did not.

### Aplastic anemia patients that did not receive alloHCT

Regarding patients that did not receive alloHCT (n=15), 14/15 were treated with rabbit ATG and cyclosporine as first line, with the addition of eltrombopag in patients treated after its approval (n=9). Only one adolescent patient, who was excluded from further analysis, did not receive ATG as first line due to comorbidities (schizophrenia) and poor performance status. The patient received only cyclosporine and steroids for a short period and succumbed due to septic shock.

**Figure 1:**
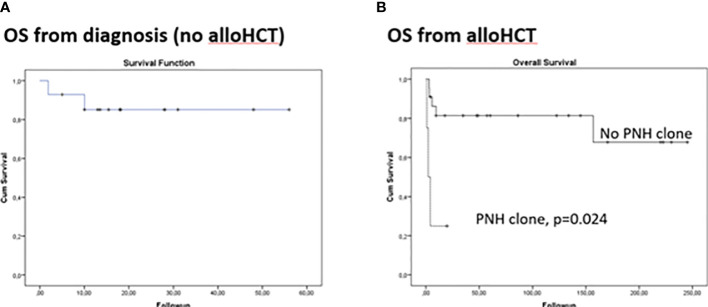
Kaplan Meier curves for overall survival in aplastic anemia patients. **(A)** From disease diagnosis in patients that did not receive allogeneic hematopoietic cell transplantation (alloHCT) **(B)** From alloHCT.

The median follow-up was 32 (1.8-56.2) months from diagnosis. Grade II-III infections were detected in 3 patients (2/3 bacteremias). One patient died early due to Ps. Aeruginosa and Fusarium infection. Another patient was diagnosed with lung cancer 2 years after treatment and succumbed to lung cancer complications. Increased viral loads of CMV and EBV (quantitative PCR) were detected in 5 patients (CMV:2, EBV:5) and gradually decreased without treatment. Other toxicities rated as grade ≥II were: hepatotoxicity (7 patients), nephrotoxicity (3 patients), and polyneuropathy (1 patient). Twelve patients became transfusion independent for RBCs in a median period of 85 days (range 10-200) and for PLTs in a median period of 62 days (range 17-187), while the absolute neutrophil count reached >500 per cubic millimeter threshold in a median of 55 days (range 9-150).PNH clones were not associated with poor response in patients not receiving alloHCT. Complete response (CR) was achieved in 7/14 (50%) and partial response (PR) in 30% of patients, with an overall response rate (ORR) of 80%. There was no significant difference in patients with or without eltrombopag. Two adult patients with PR relapsed within 40 months from initial treatment, while one pediatric patient progressed to AML and received chemotherapy. In total, 12/14 (85.7%) are alive (4-year OS: 85.1%, **Figure 1A**). It should be highlighted that these results include patients that did not undergo alloHCT either because they had no indication or no suitable donor at any time-point.

### Aplastic anemia patients that underwent alloHCT

AlloHCT was performed in 28 patients, upfront in 12/28. Bone marrow grafts (25/28) and sibling donors (19/28) were preferred when available. Engraftment was evident at day 13 post-transplant (range 12-21) for neutrophils and 39 (16-121) for platelets. Complete donor chimerism was achieved in all patients. No graft rejection or failure was observed. Two patients presented with PTLD. The first patient had central nervous system involvement with a fatal outcome, whereas the second patients had a successful resolution following rituximab administration. Five patients (18%)were diagnosed with TA-TMA according to the International Working Group (IWG) criteria as an early complication, of whom 4/5 had detectable PNH clone pre-transplant that did not require treatment (<30%). PNH clones were no longer detectable post-transplant since they had achieved full donor chimerism. TA-TMA did not resolve in 3 out of 5 patients (all with a pre-transplant PNH clone) despite best available care: cyclosporine cessation (5/5), plasma exchange (3/5) and eculizumab (1/5). Patients were followed-up for a median of 47.5 months (0.9-262.4) post-transplant, with the follow-up among survivors reaching 86.3 (6.3-262.4) months. All survivors have a complete hematologic recovery. Cumulative incidence (CI) Grade 2-4 of acute GVHD was24.3%, while CI of moderate/severe chronic GVHD was 38.6%. TRM occurred only within the first-year post-transplant. 10-year CI of TRM was 14% was attributed to GVHD or TA-TMA. 10-year OS reached 56.9% and was independently associated with PNH clones after adjusting for age and donor type (p=0.024, **Figure 1B**, **Table 2**). **Figure 1** presents comparison of OS from disease diagnosis in patients that received alloHCT (upfront or after immunosuppressive treatment) and not (p=0.877). No secondary malignancies or fatal long-term complications were documented.

**Table 2:** Cox regression analysis for Overall Survival.

	Sig.	Exp (B)	95% Confidence Intervals
**Age**	0.769	1.010	0.955-1.071
**PNH clone y/n**	0.024	0.062	0.011-0.32

PNH, Paroxysmal nocturnal hemoglobinuria; Sig, significance.

## Discussion

Our study reflects the clinical spectrum of BMF presenting with several challenges in the real-world setting. Interestingly, AA was the most common diagnosis, with PNH clones being detected in many patients. Complement inhibition treatment has revolutionized the field providing safety and efficacy in treated patients, with or without AA. AlloHCT also showed safety and efficacy. Nevertheless, the presence of a PNH clone had an independent negative impact in survival post alloHCT.

Complement inhibition with eculizumab can indeed be efficient in patients with PNH clones, regardless of the existence or not of AA (53). As reflected by our rather small patient population, approximately 25% of PNH patients develop extravascular hemolysis. Since novel complement inhibitors are under advanced clinical development, these patients may benefit from upstream complement inhibition, such as pegcetacoplan that is currently FDA approved (54). Beyond novel complement inhibitors, the role of complement inhibitors in the transplant setting remains also to be clarified.

Despite that PNH clones are common in patients with aplastic anemia, only a few recent reports have considered the presence of PNH clones (55). Previous real-world reports have not taken this issue into consideration (56, 57). Recently, DeZern et al. reported successful outcomes with eculizumab bridging before alloHCT in 8 severe/very severe (SAA) patients (58). In addition, two recent studies have also explored outcomes of patients with PNH clones in the age of eculizumab (59, 60). Although both studies presented the potential benefits of eculizumab post alloHCT in 8 and 2 patients respectively, there was no clear comparison with a historical control group that did not receive eculizumab (59, 60). This comparison would clarify the role of complement inhibition, given that alloHCT mortality in SAA patients with PNH clones has been reported at approximately 30% (61).

PNH has been traditionally considered a negative predictor after alloHCT due to the heterogeneity of clinical presentations and severe signs of hemolysis and thrombocytopenia (62). Thrombosis is the major cause of death in PNH patients (63). Despite the multifactorial nature of thrombosis in PNH, complement inhibition seem to block this vicious cycle (63). However, early prediction of thrombotic or cardiovascular risk is not yet feasible, because little is known about patients post alloHCT (64). Interestingly, the incidence of TA-TMA reported in this cohort (18%) which is similar to the incidence of 16% previously reported in all patients receiving alloHCT in our center irrespective of indication (64).

In the group of SAA patients without a PNH clone, our results were comparable to those recently reported by the Aplastic Anemia Working Party of the European Group for Blood and Marrow Transplantation with the use of sibling or unrelated donors (34). The majority of recent previous reports has documented a risk of graft rejection/failure ranging from 3% to 33% (55). In our cohort, there was no graft rejection/failure. The use of fludarabine in the conditioning regimen along with ATG might have contributed to this result (65).

Our study is limited by its retrospective nature, the rather small number of participants and experience from two centers. In contrast, it reflects the local epidemiology from Northern Greece and reports data from the pediatric and adult hematology and BMT centers located in this area. In addition, our study was performed with both sibling and unrelated donors, since expansion of the donor pool using alternative donors remains currently under consideration as an alternative option for those patients (66, 67). It should be noted however that this study was conducted according to standard operating procedures with a long-term follow-up despite difficulties during the COVID-19 period (68).

In conclusion, our real-world experience confirms that novel treatments have revolutionized the field of BMF syndromes. Nevertheless, further studies are needed to personalize algorithms in the era of precision medicine.

## Data availability statement

The datasets presented in this study can be found in online repositories. The names of the repository/repositories and accession number(s) can be found in the article/supplementary material.

## Ethics statement

The studies involving human participants were reviewed and approved by Institutional Review Board of G Papanicolaou Hospital, Exochi, Thessaloniki, Greece. Written informed consent to participate in this study was provided by the participants’ legal guardian/next of kin.

## Author contributions

All authors have made contributions to the writing and design of the manuscript, collection or analysis of the data and drafting the article or revising it critically for important intellectual content. All authors have read and agreed to the published version of the manuscript.

## Acknowledgments

The authors would like to acknowledge the valuable contribution of personnel involved in diagnosing and treating these patients. The authors would also like to thank Dr Maria Gavriilaki, an experienced writer, who revised the paper for typos and grammatical mistakes.

## Conflict of interest

EG has received honoraria from Alexion and Omeros Pharmaceuticals.

The remaining authors declare that the research was conducted in the absence of any commercial or financial relationships that could be construed as a potential conflict of interest.

## Publisher’s note

All claims expressed in this article are solely those of the authors and do not necessarily represent those of their affiliated organizations, or those of the publisher, the editors and the reviewers. Any product that may be evaluated in this article, or claim that may be made by its manufacturer, is not guaranteed or endorsed by the publisher.
